# The trend of change in cervical tumor size and time to death of hospitalized patients in northwestern Ethiopia during 2018–2022: Retrospective study design

**DOI:** 10.1002/hsr2.1121

**Published:** 2023-02-19

**Authors:** Aragaw E. Aguade, Chalachew Gashu, Tigist Jegnaw

**Affiliations:** ^1^ Statistics Department, Under Natural and Computational Science College University of Gondar Gondar Ethiopia; ^2^ Statistics Department, Under Natural and Computational Science College University of Odabultum Ethiopia

**Keywords:** Cox proportional‐hazard model, joint model, time to death, tumor size

## Abstract

**Background and Aims:**

Cervical cancer is the fourth most common cause of cancer‐related death in the world. The objective of this study was to determine factors that affect the longitudinal change of tumor size and the time to death of outpat

**Methods:**

A retrospective follow‐up study was carried out among 322 randomly selected patients with cervical cancer at the University of Gondar Referral Hospital from May 15, 2018 to May 15, 2022. Data were extracted from the patient's chart from all patients' data records. Kaplan–Meier estimator, log‐rank test, the Cox proportional‐hazard model, and the joint model for the two response variables simultaneously were used.

**Results:**

Among 322 outpatients with cervical cancer, 148 (46%) of them were human immunodeficiency virus (HIV) positive and 107 (33.3%) of them died. The results of joint and separate models show that there is an association between survival and the longitudinal data in the analysis; it indicates that there is a dependency between longitudinal terms of cervical tumor size and time‐to‐death events. A unit centimeter square rise in tumor size, corresponding to an exp(0.8502) = 2.34 times, significantly raised the mortality risk.

**Conclusion:**

The study showed that HIV, stage of cancer, treatment, weight, history of abortion, oral contraceptive use, smoking status, and visit time were statistically significant factors for the two outcomes jointly.

**Implications:**

As a result, adequate health services and adequate resource allocations are critical for cervical cancer control and prevention programs. Therefore, the government should provide adequate funding and well‐trained health professionals to hospitals to sustain screening programs with appropriate coverage of cervical cancer patient treatments.

## 1 INTRODUCTION

Cervical cancer is the fourth most common cancer among women worldwide, with an estimated incidence of 570,000 cases and 311,000 deaths, as reported in 2018.^1^ This study aims to assess the epidemiology and risk factors in developing cervical cancer. An estimated 85% of deaths from cervical cancer worldwide occur in middle‐ and low‐income countries, which have mortality rates 18 times higher than that in developed countries. Cervical cancer is known to be caused by oncogenic subtypes of the human papillomavirus (HPV). The risk factors for developing cervical cancer include: sex with multiple partners, sexual activity at an early age, having many children, use of birth control pills, smoking, low socioeconomic status, sexually transmitted diseases, oral contraceptive use, and immune disorders. Epidemiological studies have shown that the risk of developing cervical cancer and contracting genital HPV infection is influenced by a variety of factors. Thus, cervical cancer is due to a variety of additional factors working together with cancer‐associated strains of HPV.^1^

The cervical cancer epidemic in Africa is profound and complex with noninfectious and infectious risk factors and etiologic components. The cervical cancer epidemic in Africa is considered by the dual burden of noncommunicable and communicable disease,^2^ health service preventive delivery challenges,^3^, ^4^, ^5^ shortages of human resources for health,^6^ access to treatment shortages, and low cervical cancer consciousness among the health providers and population.^7^, ^8^

Many research findings show that the HPV vaccine is effective in both preventing genital warts and cervical lesions in those patients who are vaccinated; the result found that patients who are vaccinated for HPV have a lower risk of developing cervical cancer than those patients who are not vaccinated.^9^, ^10^, ^11^, ^12^ However, the Centers for Disease Control and Prevention does not recommend HPV vaccinations for people older than 26, individuals aged between 27 and 45, and who are not effectively vaccinated due to a risk for new HPV infection.^13^

Screening for cervical cancer is still essential due to the inefficient HPV vaccine protection offered. HPV vaccination protects only 70% of women against cervical cancer.^15^ Techniques of screening include: visual inspection with acetic acid (VIA) with HPV testing and Pap smear for risk of HPV types. Some of these methods, such as rapid HPV DNA and VIA testing, are preferred in developing nations due to their ease and cost of manufacture.^16^ Due to the lack of comprehensive cervical cancer treatments, early diagnosis and screening could reduce related mortality and morbidity.^17^

Artificial intelligence (AI) provides an automated diagnosis that significantly resolves the screening issue, which is verified.^18^ In recent years, AI has been used to diagnose a growing number of diseases, particularly in skin malignancies,^19^ imaging tumors,^20^ classification and detection of retinal diseases,^21^ and gynecologic cancer.^22^ AI can use sophisticated algorithms for image classification and recognition, process data autonomously, and extract features.^23^, ^24^, ^25^, ^26^

In 2020, there were 342,000 cervical cancer deaths; approximately 90% of these deaths occurred in low‐ and middle‐income countries.^26^ Programs that enable girls to receive vaccines against HPV infection and women to receive frequent screenings and appropriate care are in place in high‐income nations. Screening makes it possible to find precancerous lesions at an early stage when they are still treatable. In low‐ and middle‐income countries, cervical cancer is frequently detected only after it has progressed and symptoms appear, as access to these prophylactic practices is limited. Additionally, access to cancer treatments (e.g., cancer surgery, radiation, and chemotherapy) can be restricted, which could have an adverse impact on the condition.^27^

A total of 120,000 new instances of cervical cancer are diagnosed each year in Africa, accounting for 20% of all new cervical cancer diagnoses worldwide. Women in Africa make up a sizable portion of those who lack access to treatment and care for cervical cancer. The conventional surgical treatment for early cervical cancer, a radical hysterectomy, is not performed in many clinics and in many nations due to a lack of experience. The same is true of several African nations, with some having no radiation equipment at all. Nevertheless, some facilities are particularly well‐suited for clinical trial enrollment because they offer a higher level of care, see a lot of patients, and do so frequently. HPV and HIV prevention and screening, suitable imaging examinations, and access to adequate treatment (surgery, chemotherapy, and other forms of radiation therapy) are some of the issues faced in sub‐Saharan Africa when treating cervical cancer.^28^ Regarding total national mortality in Ethiopia, cancer accounts for roughly 5.8% of the total, except for Addis Ababa, where population‐based data is available; it is thought that there are about 60,960 new instances of cancer diagnosed each year.

In Ethiopia, cervical cancer screening (CCS) guidelines advocate a “screen‐and‐treat” method, in which women aged between 30 and 49 years are screened and treated with cryotherapy. The guidelines suggested annual screening for women who were HIV positive and three times annual screening for other women, but the screening was not consistent and was usually determined by the availability of resources.^29^

Women and their families are affected by cervical cancer anywhere in the world, but notably in places with few resources for screening, prevention and treatment. Cervical cancer affected 604,237 women worldwide in 2020, accounting for 6.5% of all female cancer cases.

In 36 low‐ and middle‐income nations, mostly in sub‐Saharan Africa, cervical cancer affects women more frequently than any other type of cancer. In 2020, cervical cancer is predicted to claim the lives of 341,843 women, 90% of whom live in underdeveloped nations with severely restricted access to services for prevention, screening, and treatment. Today, cervical cancer claims the lives of women more than childbirth does.^30^, ^31^ Ethiopia recorded 4884 deaths and 6294 new cases of cervical cancer in 2018, one of the utmost rates in the world.^1^

The investigator observed, from the University of Gondar Referral Hospital (UGRH), that the number of patients with cervical cancer being admitted to the UGRH has been rising year over year. Every year, related to cervical cancer, there are several cases of women who are dead or lost to follow‐up. This suggests that there are factors influencing both the survival status of cervical cancer patients discharged from the hospital and the progression of cancers. This calls for a change in healthcare priorities and the most recent information on the development and associated difficulties of cervical cancer in Ethiopia. Therefore, it is crucial to evaluate the variables that influence the longitudinal evolution of tumor size and the time to event (death) of outpatients with cervical cancer. Furthermore, it appears from studies that less than 10% of women in Ethiopia had CCS.^10^, ^11^, ^32^

The facts about Ethiopia mentioned above indicate that many women are at high risk for cervical cancer. While some studies have been done on cervical cancer, the majority of those done in Ethiopia concern knowledge, screening practices, and factors that predict how long cervical cancer patients will live after diagnosis. These studies range from 2008 to 2012.^10^, ^33^

The tumor size of outpatients with cervical cancer, which is one of the key factors affecting prognosis, was not highlighted in the study. In addition to this, they did not show covariates that are measured longitudinally and related to the event, and the association between the longitudinal change of tumor size and the time‐to‐event (death) outcome.

Studying the change in cervical tumor size and the mortality rates of outpatients with cervical cancer is crucial for all of the aforementioned reasons. To the best of the researcher's knowledge, no published work has recorded a joint model analysis of changes in tumor size and the time to event (death) among outpatients with cervical cancer. So, in the case of the UGRH, this study attempted to pinpoint the variables that influence the longitudinal change in cervical tumor size and the time to event (death) of outpatients with cervical cancer.

The major objective of this study was to determine the effects of the size of tumor and time to event of outpatients with cervical cancer at the UGRH; the specific objectives are as follows:

❖ To investigate factors influencing the time to event of outpatients with cervical cancer at the UGRH.
❖ To identify risk factors that affect tumor size of outpatients with cervical cancer at the UGRH.
❖ To examine the relationship between tumor size and time to event of outpatients with cervical cancer at the UGRH.

Any concerned legal body can use the output of the research to allocate proper human and material resources for cervical cancer healthcare institutions. On the other hand, healthcare institutions could provide fast and effective services for those women with high‐risk factors for cervical cancer.

This study raises awareness and education among individuals, society, the government, and any legal body about the risk factors for cervical cancer and their life expectancy. Researchers who intend to conduct research on the relationship between factors of tumor size and the time to event of women with cervical cancer might use the output of this research.

## 2 MATERIALS AND METHODS

### 2.1 Study area

The study area was the UGRH, which is located 720 km northwest of Ethiopia's capital city of Addis Ababa. The hospital has 500 beds available. The referral hospital provides for more than seven million populations in the catchment area. It provides subspecialty and specialty services, including internal medicine, surgery, pediatrics, gynecology and obstetrics, ophthalmology, psychiatry, and so on, in its outpatient and inpatient clinics. Based on registered data, there are more than 10,000 delivery services for mothers annually.^34^

### 2.2 Data

The target population of this research was all cervical cancer patients who were hospitalized between May 15, 2018 and May 15, 2022 and who met all the inclusion criteria included in this study. Patients who began cervical cancer therapy at the UGRH from May 15, 2018 to May 15, 2022 and who had at least two follow‐up visits to the department clinic for prescription refills were included in this study. Data regarding the repeated measurement of tumor size and time to death were extracted from the outpatient's chart, which contains clinical information and sociodemographics on all outpatients with cervical cancer who were followed up. In this study, outpatients with cervical cancer represent the number of patients who transfer from the hospital to another hospital or who either leave the hospital by any means or who follow the clinical treatment up to the discharge date or die before completing the treatment for any accident other than cervical cancer or those who are on treatment.

### 2.3 Methods

There are two main components of a joint model: the longitudinal outcome and the survival (time to event) outcome. The longitudinal component of the outcome variable measures the tumor size every 6 months. It is measured in centimeters squared, so it is a continuous variable that specifies a linear mixed model with random effects. The survival outcome measures the time to (event) death of outpatients with cervical cancer (0 = censored and 1 = event).

The Ethical Clearance Approval Letter was obtained from the Ethical Approval Committee, Department of Natural Computational Sciences, University of Gondar. An approval letter with a copy of the research proposal was submitted to the Department of Oncology, Gondar University Referral Hospital. Informed verbal consent was given due to the unavailability of cervical cancer outpatients at the GURH during data collection. Both institutions approved the use of informed verbal consent obtained by telephone from study participants. For deceased study participants, oral consent was obtained using the telephone number of the caregiver on the patient's chart. For those who consented to participate in the study, a retrospective review of patient charts from May 15, 2018 to May 15, 2022 provided information on outpatient sociodemographics, medical history, and treatment received.

The longitudinal submodel of the joint model was described both by the conventional linear mixed‐effects model assuming homogeneous within‐subject variance and by incorporating subject‐specific variance. Longitudinal data sets consisted of an outcome variable, *y_ij_*, and a *px*1 vector covariate, *x*_1*i*_, observed at times *t* = 1, 2, 3, …, *n_i_*, for subject *i* = 1, 2, 3, …; the subject‐specific variance was used to assess whether individuals with different tumor size variabilities have different influence on time to event (death) of outpatients with cervical cancer

(1) $$y_{\text{ij}}=\mu_{i}\left(t\right)+w_{1i}\left(t\right)+\varepsilon_{i}=\beta_{1}^{T}X_{1i}\left(t\right)+z_{i}^{T}\left(t\right)b_{i}+\varepsilon_{i},$$

where $\mu_{i}(t)=\beta_{1}^{T}X_{1i}(t)$ is the mean tumor size,  $w_{1i}(t)=z_{i}^{T}(t)b_{i}$ is a subject‐specific random effect, $\varepsilon_{i}∼N\left(0,\sigma^{2}\right)$ is a sequence of mutually independent measurement error, and $w_{1i}(t)$ is the true individual level tumor size after the overall mean trajectory and other fixed effects have been adjusted. The *b_i_* is a vector of random effects corresponding to the random effect explanatory variables *z^T^* (*t*) and modeled as IID N(0, Σ) random variables. With repeated observations, however, the correlation among values for a given subject must be taken into account. Longitudinal modeling between specific subject variations was performed to understand differences among individuals; the continuous model inside subjects' variations was employed to analyze changes over time.^35^, ^36^

The timing of an event is examined using a survival model that accounts for the time to occurrence and the censor and/or truncation. The hazard function is widely used to express the risk of an event at time *t* and obtain from the probability that the individual gets the event at time *t*, assuming that he or she survive at that time. Kaplan–Meier (KM) estimator is a nonparameter estimator of survival analysis, which is used to describe the survival of patients both graphically and numerically.

The joint model consists of two linked submodels, the measurement model for the longitudinal process and the time‐to‐event model for the survival process. The joint modeling approach was used to obtain less bias and more efficient estimates. The association between longitudinal process and survival process may arise in two ways. One is through the use of common independent factors, and the other is through stochastic dependency between *w*_1*i*_ and *w*_2*i*._

Estimation for joint models is based on the maximization of the log‐likelihood corresponding to the joint distribution of the time‐to‐event *T* and longitudinal outcomes *y*, $L\left(T_{i},\delta_{i},y_{i}|b_{i},\theta \right)$, can be specified as

(2) $$\left(\prod_{j=1}^{{\text{mi}}_{i}}\int_{f}\left(y_{i}|b_{i},\theta \right)f{\left(T_{i},\delta_{i}|y_{i},b_{i},\theta \right)}^{\delta_{i}}{\left(1-F\left(T_{i},\delta_{i}|y_{i},b_{i},\theta \right)\right)}^{\left(1-\delta_{i}\right)}\right)f\left(b_{i}\right)db_{i},$$

where $\theta ={\left(\theta_{t}^{T},\theta_{y}^{T},\theta_{b}^{T}\right)}^{T}$ denotes the full parameter vector, with *θ_t_* denoting the parameters for the event time, *θ_y_* the parameters for the longitudinal outcome, and *θ_b_* the parameters for the random effect. Assume that given the observed history, the censoring mechanism and the visiting process are independent of the true event times and future longitudinal process.

The joint density for the longitudinal response together with random effect, $p\left(y_{i}|b_{i},\theta \right)p\left(b_{i},\theta \right)$ , is given by

(3) $${\left(2\pi \sigma^{2}\right)}^{-\frac{\text{ni}}{2}}e^{\frac{\left\{-|y{-}_{i}x_{i}\beta -z_{i},b_{i}{|}^{2}\right\}}{2\sigma^{2}}}\times 2\pi^{-\text{qb}/2}\text{det}{\left(D\right)}^{-1/2}e^{{\left\{\frac{-b_{i}^{T}D^{-1}b}{2}\right\}}_{i}},$$

where *qb* denotes the dimensionality of random effects' vector and $∥x∥={\left(\sum x_{i}^{2}\right)}^{1/2}$ denotes the Euclidian vector norm. Maximization of the log‐likelihood function $l(\theta )=\sum_{i}\log p\left(T_{i},\delta_{i},y_{i},\theta \right)$ with respect to θ can be achieved using the expectation maximization algorithm or Newton–Raphson algorithm. Missing values are a frequent problem in many real‐world data settings. Multiple imputations are the most common imputation technique for addressing missing values; this paper used multiple imputations to properly account for the uncertainty in the imputed values due to missingness.^37^

It is required to compare many models using various methods to choose the parsimonious model that best fits the provided data. This paper used Akaike's information criterion, Bayesian information criterion, and likelihood ratio test.^38^

The precise nature of the joint model was selected by comparing via standard error, association parameter (*α*), and confidence interval. The association parameters quantify the magnitude of the association between the longitudinal process and the event process. It indicates that there is a dependency between longitudinal terms of cervical tumor size and time‐to‐death events. For a better understanding, we used standardized residual plots, and the hypothesis test was a two‐sided test. The data were entered and cleaned using SPSS version 20, and they were analyzed using R statistical software version 4.1.3.

## 3 RESULTS

### 3.1 Descriptive analysis

In this study, we used the guidelines for reporting statistics for clinical research in urology^39^ for the proper reporting, analysis, and interpretations of clinical research.

The change in tumor size among outpatients with cervical cancer, which was measured roughly every 6 months at the initial entry and again at the 6‐, 12‐, 18‐, 24‐, 30‐, 36‐, and 42‐month visits, is known as the longitudinal response. The sample sizes at eight time points are 322, 322, 297, 253, 208, 157, 114, and 85. Following up on patients allowed us to observe the sharply rising degree of incomplete information throughout time as a result of recovery, deaths, dropouts, missed clinic appointments, and transfers to other hospitals. The average change in tumor size was 26.16 cm^2^, with a standard deviation of 4.58 cm^2^.

The survival endpoint of interest in this study was death. Censored outpatients are those outpatients who missed contact due to lost follow‐up or transferring to another hospital. Hence, the time to death or death time in months was created by subtracting the date of first entry from the date of the last visit (death date). Thus, based on the data obtained from the UGRH, among 322 patients, 118 (36.6%) of them died, while the rest 204 (63.4%) were censored outpatients (Table 1).

**Table 1:** Descriptive results of categorical variables of outpatients' cervical cancer.

Factor	Category	Survival status		Total (%)
		Censored, 204 (63.4%)	Event, 118 (36.6%)	
Residence	Rural	73 (22.7)	52 (16.1)	125 (38.8)
	Urban	131 (40.7)	66 (20.5)	197 (61.2)
Educational level	Literate	126 (39.1)	63 (19.6)	189 (58.7)
	Illiterate	78 (24.2)	55 (17.1)	133 (41.3)
Comorbid disease	No	127 (39.4)	26 (8.1)	153 (47.5)
	Yes	77 (23.9)	92 (28.6)	169 (52.5)
History of abortion	No	151 (46.9)	38 (11.8)	189 (58.7)
	Yes	53 (16.5)	80 (24.8)	133 (41.3)
HIV	No	163 (50.6)	21 (6.4)	184 (57)
	Yes	41 (12.7)	97 (30.3)	138 (43)
Stage	Early	106 (32.9)	25 (7.8)	131 (40.7)
	Late	98 (30.4)	93 (28.9)	191 (59.3)
Oral contraceptive uses	No	93 (28.9)	18 (5.6)	111 (34.5)
	Yes	96 (29.8)	115 (35.7)	211 (65.5)
Histology type	Adenocarcinoma	153 (47.5)	21 (6.5)	174 (54)
	Squamous cell	51 (15.8)	97 (30.2)	148 (46)
Treatment	Radiotherapy	81 (25.2)	37 (11.5)	118 (36.7)
	Chemotherapy	82 (25.5)	43 (13.3)	125 (38.8)
	Surgery	41 (12.7)	38 (11.8)	79 (24.5)
Smoking status	No	186 (57.76)	52 (16.14)	238 (73.9)
	Yes	18 (5.6)	66 (20.5)	84 (26.1)

Abbreviation: HIV, human immunodeficiency virus.

### 3.2 Log‐rank test and KM estimates

**Figure 1:**
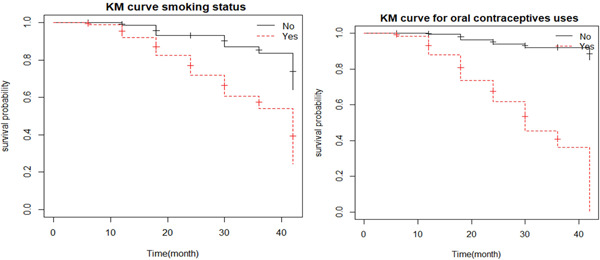
Kaplan–Meier (KM) plot of oral contraceptive use and smoking status of patients, 2018–2022.

The KM curve for the smoking status of cervical cancer patients shows that patients who do not smoke have a higher chance of surviving than patients who smoke. The survival probability of outpatients who do not take oral contraceptives is greater than that of outpatients who do, according to the plot of the KM curve for oral contraceptive use. Plots of the KM estimates for the two selected categorical covariates: oral contraceptives and smoking status are displayed in Table 2 and the remaining categorical variables are presented in Figure 1.

**Table 2:** Log‐rank test of categorical independent variables.

Covariate	*df*	*χ* ^2^	*p* Value
Treatment	2	8.6	0.01
Residence	1	0.8	0.4
HIV	1	110	<0.0001
Stage	1	11.8	<0.0001
Oral contraceptive uses	1	93.3	<0.0001
Histology type	1	65.1	<0.0001
Smoking	1	29.9	<0.0001
Comorbid disease	1	41.6	<0.0001
History of abortion	1	25.5	<0.0001
Education	1	1.8	0.2

Abbreviation: HIV, human immunodeficiency virus.

To assess the significance of differences across various factors, log‐rank tests were performed on all categorical variables. According to the null hypothesis, there is no discernible difference between the rates of survival for various categories of categorical variables. The log‐rank tests in Table 2 revealed that, at the 5% level of significance, there is no difference between groups of residence and education in terms of the time to death. Death rates among the study groups differ significantly for other categorical factors.

Table 3 indicates that the proportional‐hazard (PH) assumption states that the hazard ratios are constant across time. Therefore, the risk of failure must remain consistent regardless of how long patients have been observed. This hypothesis was tested using Schonfeld's residual and a GLOBAL test. To test the assumption of the Cox proportional hazard (PH) model, we first fitted a univariable Cox PH model. The univariable Cox PH regression models are fitted for each covariate to find factors affecting patients' survival from cervical cancer before moving on to more complex models. The problem with the single covariate approach is that it ignores the potential that a collection of variables, each of which has only a very weak relationship to the outcome, can come together to significantly predict the outcome.

**Table 3:** Cox proportional‐hazard assumption of outpatients with cervical cancer.

Covariate	*χ* ^2^	*df*	*p* Value
Education	0.3629	1	0.547
Treatment	0.0421	2	0.979
Baseline weight	1.1559	1	0.282
Histology type	0.6807	1	0.409
Stage	0.8801	1	0.348
HIV	0.9662	1	0.326
Oral contraceptive use	1.0028	1	0.317
Comorbid disease	2.7651	1	0.096
History of abortion	0.1897	1	0.663
Smoking	0.0772	1	0.781
GLOBAL	6.2570	11	0.86

Abbreviation: HIV, human immunodeficiency virus.

Based on the univariable results, the building blocks for a multivariable Cox model are as follows: baseline weight of patients; smoking status; histology type; HIV, stage, treatment; oral contraceptive use; education; history of abortion; and presence of comorbid disease. The estimated values, standard errors, and *p* values for each variable are given (Supporting Information: Appendix C.1). By the purposeful variable selection method, first testing the significance of each variable at a 25% level of significance, then by only the variables significant at this level could fit the multivariable Cox PH model.

In Table 3, the Schoenfeld residuals and survival time do not have a statistically significant correlation. This suggests that the 0.05 threshold of significance for the GLOBAL test's “*p* < 0.86” is not significant and that all the factors satisfy the proportionality assumption. Therefore, the Cox model's PH assumption is not broken, according to this. The proportionality presumption is valid because it is presumed that the hazard ratios will stay unchanged throughout time. Therefore, for the survival part of this study, the Cox PH model was used.

**Figure 2:**
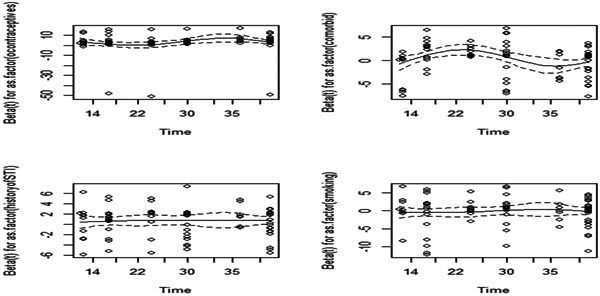
Scaled Schoenfeld residual plot for some selected predictor variables, University of Gondar Referral Hospital, 2018–2022.

The graphical inspection in Figure 2 shows that there is no pattern with respect to time. The PH assumption appears to be supported by covariates such as weight, comorbidity, smoking, history of abortion, and oral contraceptive use. Since the PH assumption had not been violated, the Cox PH model was employed to assess the time‐to‐event data.

In Table 4, in the Cox regression model analysis, factors that had a statistically significantly positive association with time to death were oral contraceptive use, HIV, late‐stage, surgery, squamous cell carcinoma, comorbid disease, and a history of abortion.

**Table 4:** Cox PH model estimates of outpatients with cervical cancer.

Parameter	Estimate	SE	HR	95% CI		*p* Value
				Lower	Upper	
Baseline weight	−0.9458	0.01621	0.9552	−0.9854	−0.926	0.005
Treatment (ref. = radiotherapy)						
Chemotherapy	0.38100	0.23337	1.4638	0.2265	1.6127	0.10
Surgery	0.7372	0.25234	2.6478	0.6147	0.9418	0.0001
Education (ref. = literate)						
Illiterate	−0.0324	0.54176	0.4808	−0.1663	1.3902	0.18
Histology (ref. = adenocarcinoma)						
Squamous cell	0.8563	0.32557	2.35	0.2244	0.9739	0.008
Stage of cancer (ref. = early)						
Late	0.62240	0.27301	1.863	0.3143	0.9164	0.022
HIV (ref. = no)						
Yes	0.38100	0.42206	1.46	0.0293	0.9207	0.0001
Oral contraceptive uses (ref. = no)						
Yes	1.38048	0.67544	3.59837	1.1747	5.296	0.0001
Comorbid disease (ref. = no)						
Yes	0.38502	0.27059	1.4697	0.1800	0.9411	0.024
History of abortion (ref. = no)						
Yes	0.72158	0.24687	2.0577	0.484	0.882	0.003
Smoking status (ref. = no)						
Yes	−0.1774	0.31981	0.8374	−0.874	1.363	0.58

Abbreviations: CI, confidence interval; HIV, human immunodeficiency virus; HR, hazard ratio; PH, proportional hazard; ref., reference category.

In Table 5, the finding demonstrates that the change in tumor size was statistically significant correlated with the predictors of visit time, treatment, weight, cancer stage, HIV, oral contraceptives, history of abortion, and smoking status. Additionally, the results show that the predictors of surgery, oral contraceptives, cancer stage, HIV, weight, history of abortion, and comorbid diseases had a highly statistically significant relationship between the risk of death of outpatients with cervical cancer and the survival process. Tumor size and risk of death of outpatients with cervical cancer were simultaneously influenced by HIV, cancer stage, treatment, weight, history of abortion and oral contraceptive use. In the survival sub‐model under joint analysis, the estimate of the association parameter (α) was statistically significantly different from zero, which, in line with our findings, gives strong support for a relationship between the two outcomes. The estimation of the association parameter results indicates that tumor size has a statistically significant positive relationship with the risk of death in outpatients with cervical cancer.

**Table 5:** Joint model estimates of outpatients with cervical cancer

	Longitudinal process					Survival process				
	Estimate	SE	95% CI			Estimate	SE	95% CI		
Parameter			Lower	Upper	*p* Value			Lower	Upper	*p* Value
Intercept	4.4716	0.1519	4.168	4.823	<**0.0001**	5.3447	0.4715	4.421	6.269	<**0.0001**
Education (ref. = literate)										
Illiterate						−0.0305	0.1667	−0.357	0.2963	0.8550
Treatment (ref. = radiotherapy)										
Chemotherapy	0.1624	0.0819	0.0085	0.223	**0.0474**	−0.0270	0.0712	−0.1666	0.1126	0.7044
Surgery	0.1841	0.0931	0.0192	0.317	**0.048**	0.2343	0.0739	0.19	0.439	**0.0021**
Weight	−0.0098	0.0043	−0.0152	−0.00098	**0.03**	−0.2072	0.0046	−0.34	−0.299	**0.0306**
Histology type (ref. = adenocarcinoma)										
Squamous cell	−0.0540	0.1096	−0.269	0.1507	0.622	0.1299	0.0992	−0.0645	0.3243	0.1902
Stage of cancer at diagnosis (ref. = early)										
Late	0.2226	0.0947	0.0469	0.4083	**0.0188**	0.1721	0.0144	0.066	0.473	**0.0415**
HIV (ref. = no)										
Yes	0.4360	0.1173	0.2161	0.666	**0.0002**	0.4636	0.129	0.3914	1.0157	**0.0405**
Oral contraceptive use (ref. = no)										
Yes	0.2920	0.1117	0.082	0.4109	**0.0089**	0.6094	0.2084	0.201	4.018	**0.0034**
Comorbid (ref. = no)										
Yes	0.0856	0.0913	−0.0903	0.164	0.3483	0.512	0.0834	0.475	1.135	**0.0107**
History of abortion (ref. = no)										
Yes	0.2376	0.0933	0.077	0.4106	**0.0109**	0.70	0.0797	0.562	0.931	**0.0416**
Smoking status (ref. = no)										
Yes	0.3003	0.1137	0.180	0.513	**0.0083**	−0.0037	0.0996	−0.199	1.192	0.9706
Visit time	−0.0084	0.0016	−0.0115	−0.00527	**<0.0001**					
Association (*α*)						0.8502	0.0650	0.1227	1.6777	**0.0001**
Random effect, SD (estimate)										
Intercept (*b*_0*i*_)	0.5034									
Visit time (b_1*i*_)	0.0116									
Residual (*ε_i_*)	0.2825									
Correlation (*b*_0*i*,_ *b*_1*i*_)	0.4250									

*Note*: Bold values indicate that the *p*‐values are significant.

Abbreviations: CI, confidence interval; HIV, human immunodeficiency virus; ref., reference category.

A unit centimeter square rise in tumor size, corresponding to an exp(0.8502) = 2.34 times, significantly raised the mortality risk. The joint model's results in Table 5 show that there is a statistically significant relationship between the change in tumor size and mortality risk due to cervical cancer.

The parameter estimates for the individual and joint models are roughly similar but not identical; therefore, we must compare the standard errors of the two models separately and together for relevant predictors. Table 5 demonstrates that the joint model has lower standard errors for all significant predictors when compared to the separate models. The significant predictors in the joint model's survival submodel were statistically significant relationships with the risk of death, just like they were in the separate survival model in Table 5, but the joint model's standard error was much lower. The significant variables in the longitudinal submodel model with a joint model have a smaller standard error than the significant factors in the separate longitudinal model. In terms of low standard errors, the joint model fared generally better for this investigation than a separate model. As shown in Table 5, the estimation of the association coefficient in the survival sub‐analysis of the joint model was not equal to 0. This suggests that two outcome variables are correlated and this helps us to make valid inferences and conclusions; the joint model was better to fit the data, and there is a statistically significant and nonignorable difference due to the measurement error of tumor size.

In Table 5, both joint models and separate analyses use a random effect. This demonstrates that the variation of random intercepts was greater than the variance of random slopes, pointing to a more significant baseline difference. The correlation coefficient between the random intercept and the random slope is also shown in the two model results. This demonstrates that this shows that slopes and random intercepts are correlated, demonstrating that each individual's slope and intercept are correlated with one another. As a result, the correlation between slopes and intercepts was 0.4250, indicating a positive correlation between intercept and slope of linear time, and the variability within patients was 0.2825 in the joint model. The variability between patients in intercept was therefore 0.5034, and the variability between patients in slope was 0.0116. About 76% of the variation in tumor size that is not explained by predictor variable is attributable to subjects.

The estimated coefficient value of the fixed effect intercept, which can be calculated by removing all covariates from the model, was calculated as 4.5716, meaning that the anticipated change in tumor size for the outpatient with cervical cancer was 4.5716 cm^2^.

The estimated tumor size change of cervical cancer patients was statistically significant decrement by 0.0098 cm^2^ for a 1 kg rise in weight, all other factors remaining constant.

Keeping all other factors constant, the estimated tumor size changes in HIV‐positive cervical cancer patient by 0.4360 cm^2^ higher than HIV‐negative outpatients with cervical cancer. While controlling for all other factors, the estimated tumor size change of outpatients with cervical cancer in the late stage was substantially higher by 0.2226 cm^2^ than that of early stage outpatients with cervical cancer at an early stage.

The predicted tumor size change in outpatients with cervical who received chemotherapy treatment was substantially greater by 0.1624 cm^2^ compared to outpatients with cervical cancer who received radiotherapy treatment, with all other factors being constant. The expected tumor size change in outpatients with cervical who had surgery treatment was significantly higher by 0.1841 cm^2^ compared to the outpatients with cervical cancer who had radiotherapy treatment alone, all other factors being constant.

When all other factors were held constant, the estimated tumor size change of outpatients with cervical who used an oral contraceptive was considerably greater by 0.2920 cm^2^ than it was for outpatients with cervical who did not use an oral contraceptive. When all other factors were held constant, outpatients with cervical cancer who had a history of abortion had an estimated tumor size change that was considerably greater by 0.2376 cm^2^ than those with no such history.

When all other factors were held constant, the estimated tumor size change of outpatients with cervical with smokers was 0.3003 cm^2^, which was significantly larger than it was for outpatients with nonsmokers. The average progression of tumor size from a longitudinal process was significantly negatively affected by visit time in this investigation. The average tumor size of outpatients with cervical would decrease by 0.0084 if the follow‐up period was extended by one unit. This suggests that the individuals who received longer follow‐ups experienced a specific decrease in tumor size. The severity of the condition rises as the number of monthly follow‐up visits decreases.

The estimated hazard ratio of weight for cervical cancer patients exp(−0.2072) = 0.8, which indicates that the risk of death for a 1 kg increase in weight was decreased by 20% than the risk of death for a 1 kg decrease in weight, all other factors remaining constant.

The estimated hazard ratio of death for cervical cancer patients who had surgery treatment relative to cervical cancer patients who had radiotherapy treatment was exp(0.2343) = 1.264. The risk of death of outpatients with cervical cancer who had surgery treatment was increased by 26.4% compared to the risk of death for cervical cancer patients who had radiotherapy treatment, keeping all other factors constant.

The estimated hazard ratio of death of outpatients with cervical cancer who had a late stage relative to cervical cancer patients who had early stage was exp(0.1721) = 1.188. The risk of death of outpatients with cervical cancer who had a late stage was increased by 18.8% compared to the risk of death of outpatients with cervical cancer who had an early stage, keeping all other factors constant.

The estimated hazard ratio of death of outpatients with cervical cancer who had HIV positive relative to outpatients with cervical cancer who had HIV negative was exp(0.4636) = 1.59. The risk of death of outpatients with cervical cancer who had HIV positive was increased by 59% compared to the risk of death of outpatients with cervical cancer who had HIV negative, keeping all other factors constant.

The estimated hazard ratio of death of outpatients with cervical cancer who used an oral contraceptive compared to outpatients with cervical cancer who did not use an oral contraceptive was exp(0.6094) = 1.84. The risk of death of outpatients with cervical cancer who used an oral contraceptive was increased by 84% compared to the risk of death of outpatients with cervical cancer who did not use an oral contraceptive.

The estimated hazard ratio of death of outpatients with cervical cancer with a comorbid disease compared to outpatients with cervical cancer without comorbid disease was exp(0.70) = 2.01, meaning that the risk of death of outpatients with cervical cancer with a comorbid disease was 2.01 times higher than the risk of outpatients with cervical cancer with no comorbid disease, all other factors being constant.

The estimated hazard ratio of death for cervical cancer patients with an abortion history compared to outpatients with cervical cancer without an abortion history was exp(0.512) = 1.67, which means that, when all other factors were held constant, the risk of death was increased by 67% of outpatients with cervical cancer with an abortion history.

**Figure 3:**
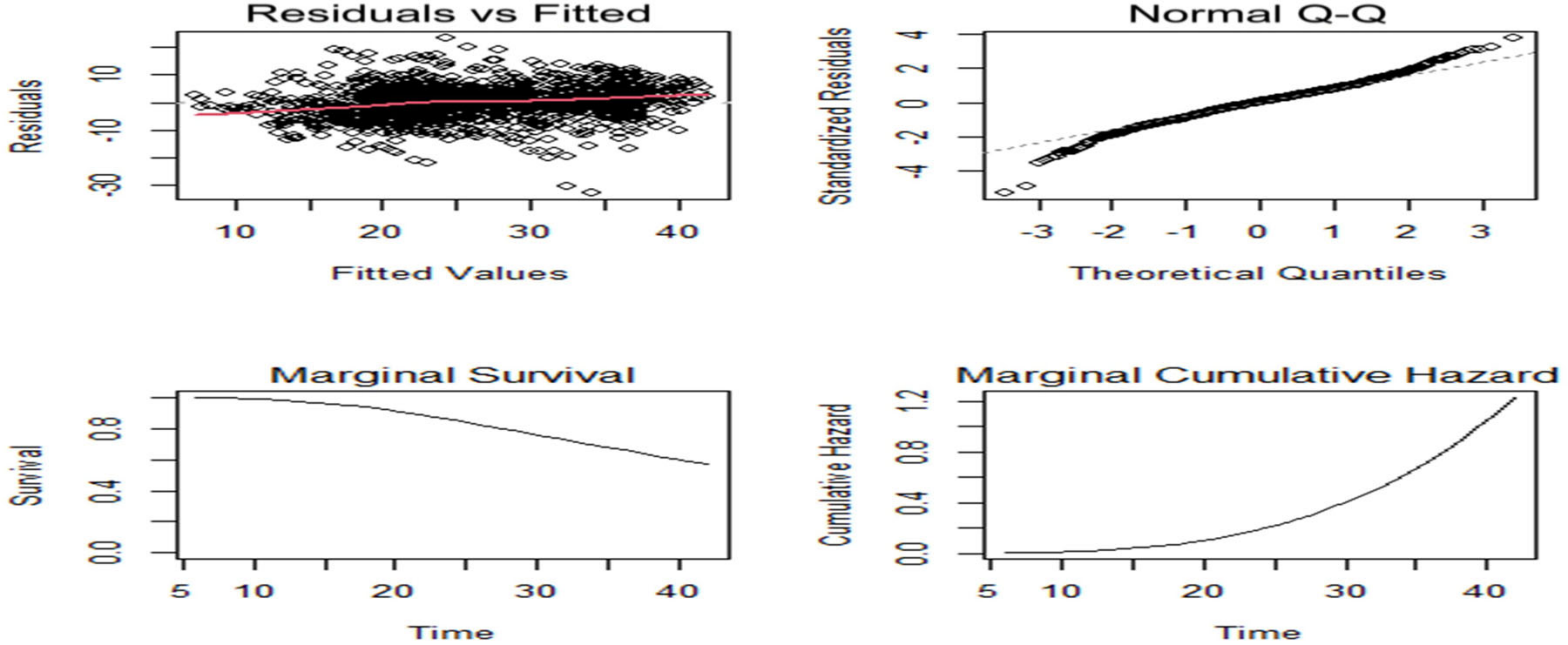
Diagnostics plot for fitted Joint model of outpatients with cervical cancer, University of Gondar Referral Hospital, 2018–2022.

To validate the assumptions behind the longitudinal submodel and the survival submodel, we usually use common types of residual plots. The fitted joint model's default diagnostic plots for cervical cancer patients undergoing follow‐up from 2018 to 2022 are displayed in Figure 3. In the top left panel, the longitudinal submodel's subject‐specific residuals and their associated fitted values are displayed. There is little doubt that the residuals closely resemble the fitted line. The constant variance assumption is supported by the plot's lack of any identifiable systematic structure. In the upper right panel, a typical Q–Q plot shows the standardized residuals of the longitudinal process. The points either fall far from the straight line or are dispersed. As a result, we can say that the longitudinal submodel's assumption of the error term's normality was met.

The bottom left plot of the marginal survival versus time indicates that the survival probability is decreasing as time increases. According to the bottom right plot of the marginal cumulative hazard plot, the cumulative hazard is increasing as the time increases.

**Figure 4:**
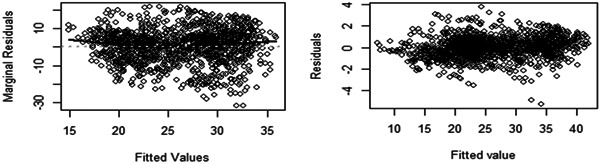
Diagnostics plot for longitudinal submodel from the joint model, University of Gondar Referral Hospital, 2018–2022.

To assess the overall goodness‐of‐fit test of the longitudinal submodel of the joint model, a scatter plot of the two types of residual conditional (subject‐specific) and average population (marginal) versus the corresponding fitted values can be employed. Figure 4 below compares the marginal residuals and subject‐specific to the fitted value of the longitudinal submodel model. Homoscedasticity and normality assumptions can be verified using these residuals, which also predict conditional errors. Because subject‐specific residuals were clustered around zero and there is no consistent trend in subject‐specific residuals versus fitted, the model assumptions provide a good fit for the data. The marginal residual versus fitted values graphics are concentrated with 0. It shows that the longitudinal submodel in the joint model assumptions was satisfied and successfully fitted. Using Q–Q and histograms, the longitudinal model's initial assumptions about the longitudinal outcome's normality were evaluated. Based on the above‐mentioned mentioned graph, it can be shown that the real tumor size satisfies the assumption of normality without transformation. As a result, we used the original actual data.

**Figure 5:**
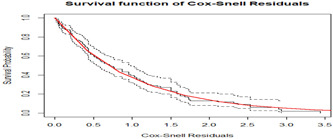
Diagnostics plot for survival submodel from q joint model, University of Gondar Referral Hospital, 2018–2022.

To check the PH assumption of the Cox PH model of the survival submodel, the Schoenfeld residuals and the formal statistical test were displayed. The Cox PH assumption for the survival submodel of the time to death among outpatients with cervical cancer was checked graphically by Schoenfeld residuals. Systematic departures from the horizontal line indicate Cox PH model was not appropriate, since it assumes that the estimates of the predictors do not vary much over time. In the plots of Schoenfeld residuals against time, all the significant covariates on the time to death of outpatients with cervical cancer showed randomness and the smooth curve was approximately horizontal straight line, this result was an implication of zero slope. So, all significant predictors included in the survival submodel had zero slopes (the curve exhibits no departures from the origin). Further from graphical tests, the global test was conducted using the covariates in the model. Since the global test was statistically insignificant at the 5% level of significance, the proportionality assumption of the Cox PH model was satisfied. Figure 5 demonstrated model diagnosis based on Cox–Snell residuals with 95% CI for the KM estimate of the Cox–Snell residuals along the red line. This result indicated that the survival process model fitted the data well.

## 4 DISCUSSION

The objective of this investigation was a joint model of longitudinal tumor size change and time to event (death) of outpatients with cervical cancer at the UGRH, Gondar, Ethiopia.

In our results, the variable weight, stage, contraceptive use, comorbid disease, HIV, treatment, and history of abortion had a significant effect on tumor size and time to event (death) of outpatients with cervical cancer at the UGRH jointly.

For this study, based on the data obtained from the UGRH, 118 (36.6%) patients died. This is lower compared to a research article conducted in Ethiopia; among 346 patients, 223 (64.4%) of them died.^40^

Accordingly, the stage of the disease also had a significant association with both risk of mortality and tumor size of outpatients. The risk of mortality of outpatients with cervical cancer who had a late stage was increased by 18.8% compared to the risk of mortality of outpatients with cervical cancer who had an early stage. This outcome is consistent with research done in Ethiopia.^41^

According to the findings of the study, oral contraceptive use was a highly significant predictor of tumor size and time to event (death) of outpatients with cervical cancer in the UGRH. Outpatients with cervical cancer who used oral contraceptives had an increase of 84% compared to the mortality risk of those who did not use oral contraceptives. This outcome coincides with the result of Smith et al.^42^

According to the findings of the study, the presence of HIV was a strong predictor of both the tumor size and the risk of death of outpatients with cervical cancer at the UGRH. The risk of mortality of outpatients with cervical cancer with HIV was increased by 30% compared to the risk of mortality for outpatients with cervical cancer without HIV. This happens because cervical cancer makes viral load counts higher and they are more likely to die. This result coincides with Gurmu^43^ and Seifu et al.^40^

The results of this study indicated that the history of abortion was a significant predictive factor for both tumor size progression and time to event (death) of outpatients with cervical cancer at the UGRH. The risk of death of outpatients with cervical cancer who had a history of abortion was increased by 67% compared to the risk of death of outpatients with cervical cancer who had no history of abortion. This result coincided with Gurmu.^43^

The results of this study suggested that comorbid disease was a significant predictive factor for the survival time of the patients. Outpatients with cervical cancer without comorbid diseases had a longer survival time than those with comorbid diseases. The risk of death for cervical cancer patients who had a comorbid disease was 2.01 times higher than the risk of death for cervical cancer patients who did not have a comorbid disease. This result coincided with that of Mebratie et al.^44^

In our result, education and histology type have an insignificant effect on both tumor size and time to death. This is in contradiction with Mebratie et al.^44^ and Getahun et al.,^45^ respectively.

## 5 CONCLUSION

In this research, we verified a longitudinal model for tumor size of outpatients with cervical cancer, a Cox PH model for time to event (death), and a joint model for the two outcomes variables together were used.

There is no discernible difference in the time to event (death) between the groups of residency and education, according to the results of the KM curve and log‐rank test. The KM curve showed that nonsmokers had a higher likelihood of surviving than smokers. Similar to this, the KM curve plot for contraceptive use demonstrates that outpatients who do not use oral contraceptives have a higher chance of living than those who do. The joint model performs better at pinpointing the variables associated with the concurrent measurements of tumor size and time to event (death) based on the standard error, CI, and association value.

Based on the results, the variables HIV, oral contraceptive use, stage of cancer, weight, history of abortion, and treatment were statistically significant factors of tumor size and time to death of outpatients with cervical cancer.

The variable longitudinal measure of tumor size has an association with time to death; hence, the joint model is the recommended one for such kinds of correlated data.

Based on the findings, we suggest some recommendations to the concerned body: to address the problems, especially vulnerable outpatients with cervical cancer who used oral contraception, who were HIV positive, and who have a comorbid disease, the health professionals should have continuous health checkups and timely medical care so as to minimize the risk of deaths. Both governmental and nongovernmental organizations should allocate adequate funding for cervical cancer treatments to promote women to get access to CCS, detection, and also full medication purposes to reduce the risk factor of cervical cancer. Health professionals give attention when a patient's estimated tumor size is increased through follow‐up, oral contraceptive use, HIV positive, history of abortion, or late stage. Finally, the researcher suggested that this work can be expanded in the future by integrating significant variables that were not examined in this particular study.

## 6 LIMITATIONS

The main limitation of the study was that some predictors such as the number of sexual partners, age at first sexual intercourse, and others were not available due to the retrospective nature of the data.

## AUTHOR CONTRIBUTIONS

**Aragaw E. Aguade**: Conceptualization; data curation; formal analysis; investigation; methodology; project administration; resources; supervision; visualization; writing—original draft; writing—review and editing. **Chalachew Gashu**: Conceptualization; data curation; formal analysis; investigation; methodology; project administration; resources; supervision; visualization; writing—original draft; writing—review and editing. **Tigist Jegnaw**: Conceptualization; data curation; formal analysis; investigation; methodology; supervision; writing—review and editing.

## CONFLICT OF INTEREST STATEMENT

The authors declare no conflict of interest.

## ETHICS STATEMENT

All methods are performed according to the relevant regulations and guidelines of the journal. The ethical letter was taken from the University of Gondar, College of Natural and Computational Sciences Ethical Committee with reference no. CNCS/10 976/11/05/2022 and the committee approved the study. The University of Gondar Referral Hospital ethical review board waived the intended consent of individual patients on February 3, 2014 because we used secondary data from hospital patients' medical cards (charts).

## TRANSPARENCY STATEMENT

The lead author, Aragaw Eshetie Aguade, affirms that this manuscript is an honest, accurate, and transparent account of the study being reported; that no important aspects of the study have been omitted; and that any discrepancies from the study as planned (and, if relevant, registered) have been explained.

## Supporting information

Supplementary information.

## Data Availability

The authors confirm that the data supporting the findings of this study are not publicly available because the data were used for an another analysis. However, data are available from the corresponding author on reasonable requests. The guarantor has full access to all the data in this study and takes complete responsibility for the integrity of the data and the accuracy of the data analysis.
