# Supplementary material for: The trend of change in cervical tumor size and time to death of hospitalized patients in northwestern Ethiopia during 2018–2022: Retrospective study design
Source: Health Sci Rep. 2023 Feb 19;6(2):e1121. doi: 10.1002/hsr2.1121 (PMC9939582; doi:10.1002/hsr2.1121)
Supplement: Supplementary file 1 — Supplementary information. [file HSR2-6-e1121-s001.pdf]

1. Appendix

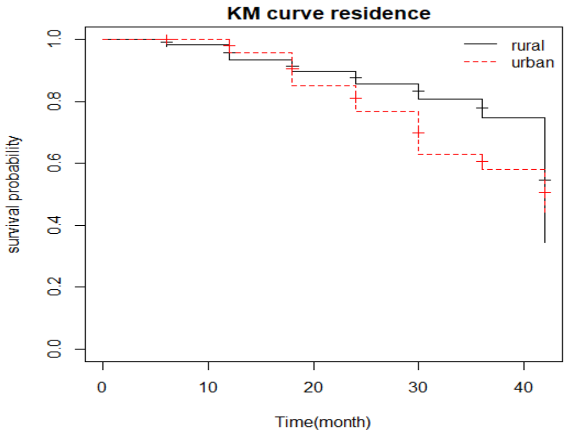
KM curve residence
rural
urban
survival probability
0.0
0.2
0.4
0.6
0.8
1.0
0
10
20
30
40
Time(month)

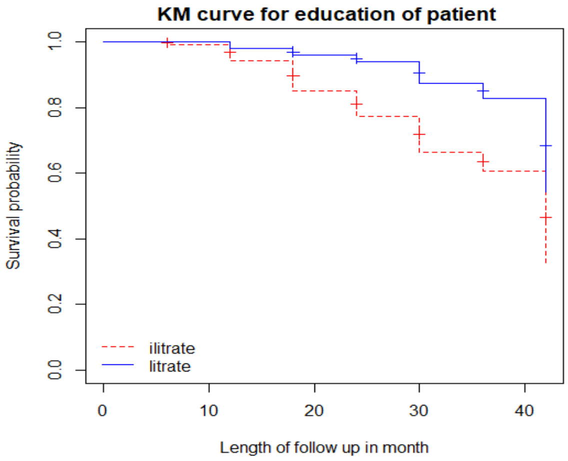
KM curve for education of patient
ilitrate
litrate
Survival probability
0.0
0.2
0.4
0.6
0.8
1.0
0
10
20
30
40
Length of follow up in month

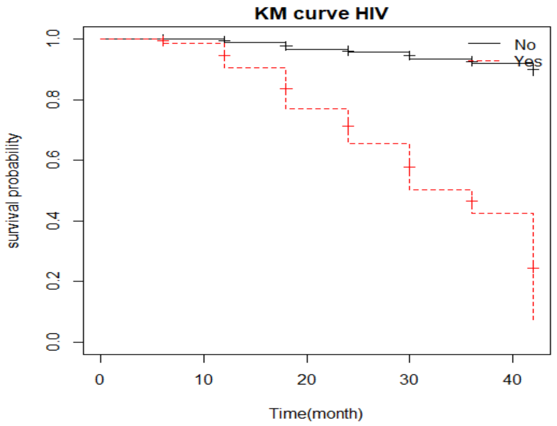
KM curve HIV
No
Yes
survival probability
0.0
0.2
0.4
0.6
0.8
1.0
0
10
20
30
40
Time(month)

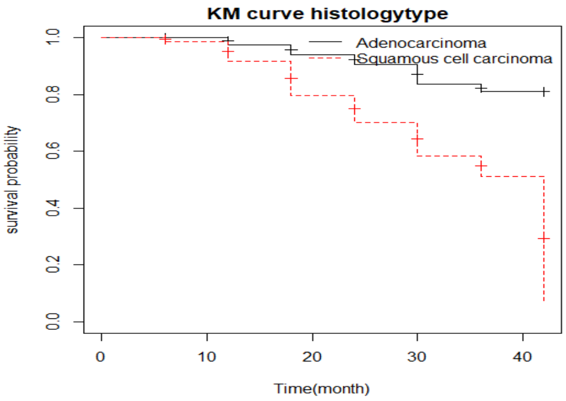
KM curve histologytype
Adenocarcinoma
Squamous cell carcinoma
survival probability
0.0
0.2
0.4
0.6
0.8
1.0
0
10
20
30
40
Time(month)

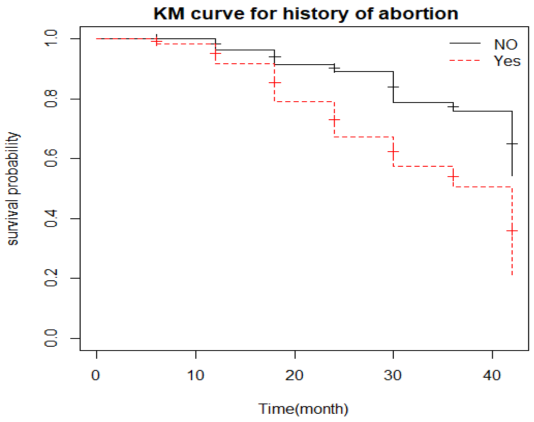
KM curve for history of abortion
NO
Yes
survival probability
0.0
0.2
0.4
0.6
0.8
1.0
0
10
20
30
40
Time(month)

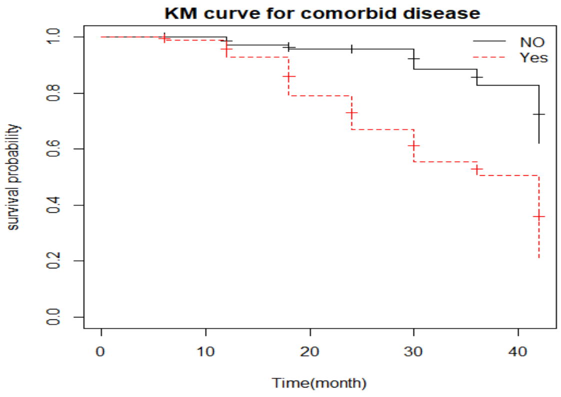
KM curve for comorbid disease
NO
Yes
survival probability
0.0
0.2
0.4
0.6
0.8
1.0
0
10
20
30
40
Time(month)

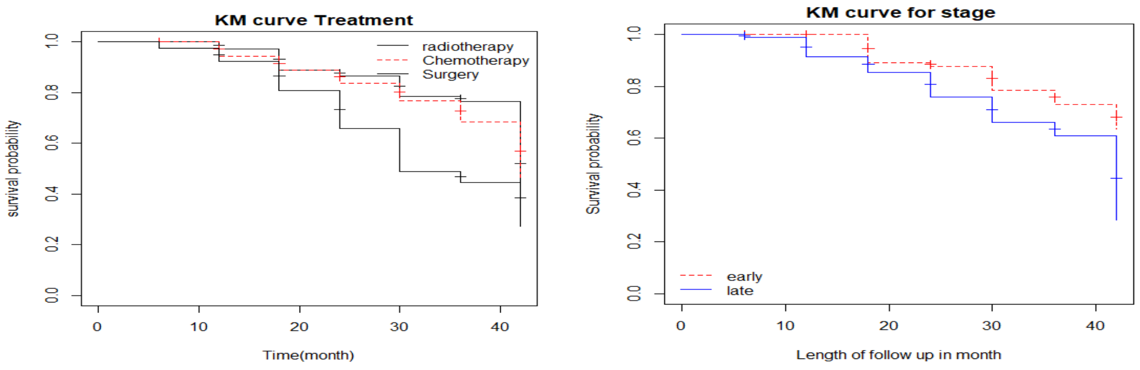

Appendix A.1 Kaplan-Meier Survival Curve for different groups

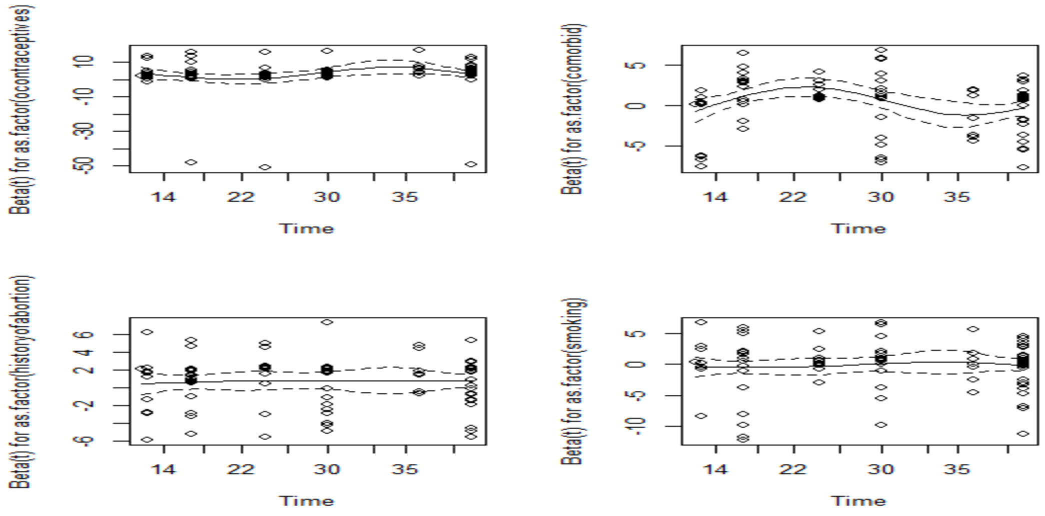

Appendix A.2 PH assumption tests by Schoenfeld residuals for significant predictors

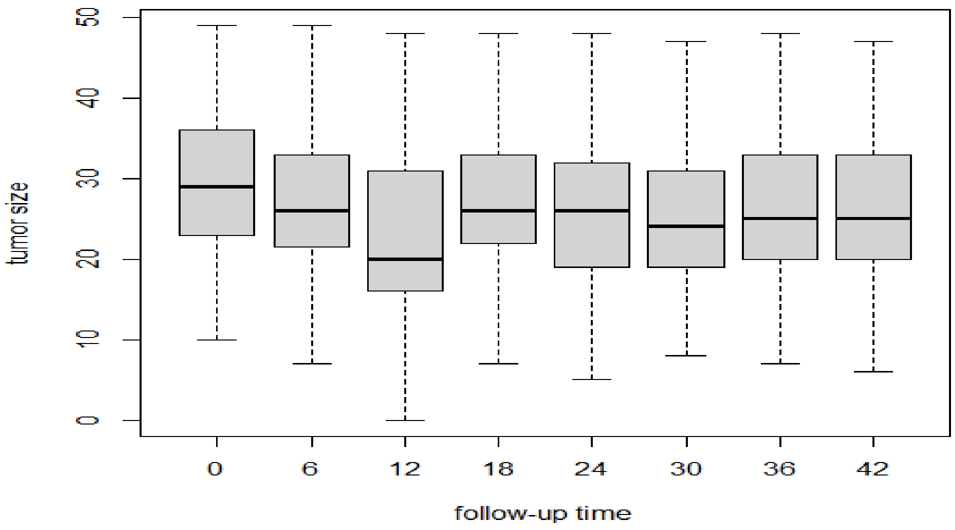

Appendix B.1 Box plot for tumor length

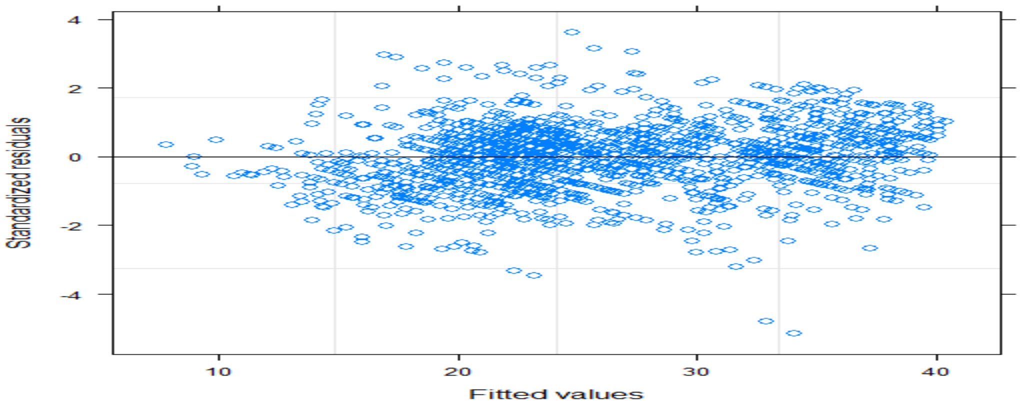

Appendix B.2 Standardized residual vs. fitted value of longitudinal tumor

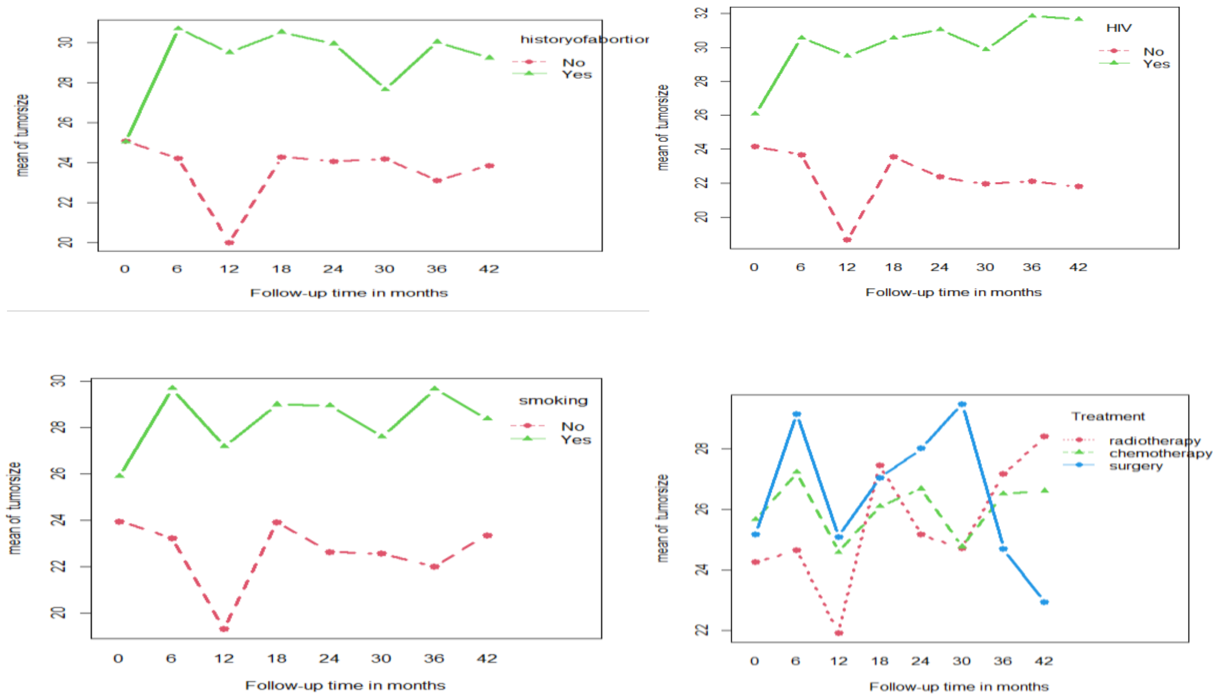

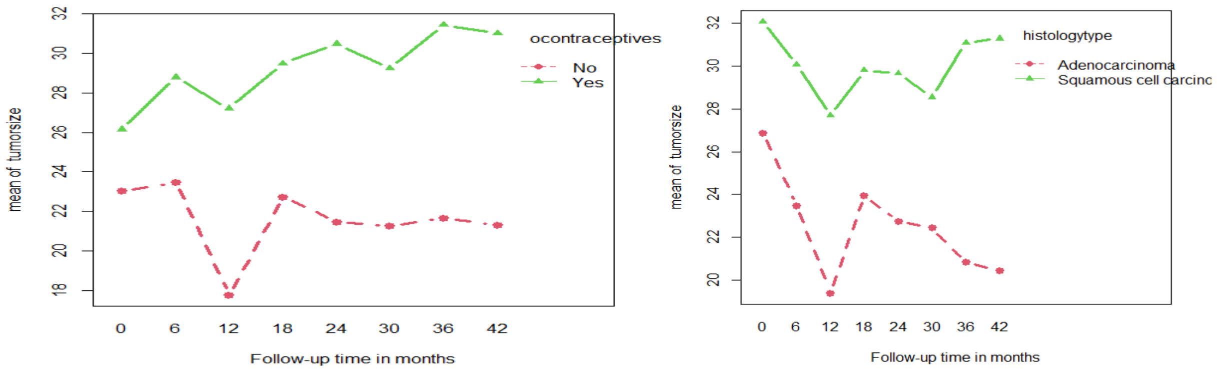

Appendix B.3 the mean profile of categorical variables

Covariate	estimate	std.err	95%CI of CC		p-value
			lower	Upper	
residence(ref=rural)					
urban	0.1136	0.1873	0.7761	1.617	0.544
HIV(ref=No)					
Yes	2.6900	0.3201	7.867	27.59	<2e-16 **
Stage of cancer at diagnosis (ref=early)					
late	0.7739	0.2258	1.393	3.375	0.00061 **
o.contraceptives (ref=No)					
Yes	3.6381	0.5873	12.03	120.2	5.84e-10 **
Treatment (ref=radiotherapy)					
Chemotherapy	-0.1461	0.2250	0.5560	1.343	0.5160
Surgery	0.4244	0.2316	0.9709	2.407	0.0669
smoking (ref=No)					
Yes	1.2053	0.2366	2.099	5.307	3.51e-07 **
History of abortion (ref=No)					
Yes	0.9603	0.1975	1.774	3.847	1.16e-06 **
comorbid (ref=No)					
Yes	1.3259	0.2224	2.435	5.823	2.5e-09 **
Educ (ref=literate)					
Illiterate	-0.6228	0.5105	0.1972	1.459	0.222

histology type (ref= Adenocarcinoma)					
Squamous cell	1.8569	0.2447	3.964	10.35	3.26e-14 **
Age	0.0003112	0.0082609	0.9842	1.017	0.97
weight	-0.06705	0.01462	0.9087	0.9623	4.51e-06 *

Appendix C.1 univariable analysis of Cox-Proportional Hazard Model
